# Cerebrospinal fluid flushing as a means of neuroprotection

**DOI:** 10.3389/fnins.2023.1288790

**Published:** 2023-12-13

**Authors:** Martin A. Dufwenberg, Alec R. Garfinkel, Mark Greenhill, Armand Garewal, Michael Craig Larson

**Affiliations:** ^1^Department of Radiology, University of Arizona, Tucson, AZ, United States; ^2^Department of Radiology, California Northstate University, Elk Grove, CA, United States; ^3^HCA Florida Brandon Hospital, Brandon, FL, United States; ^4^Department of Radiology, University of California, Davis, Davis, CA, United States

**Keywords:** cerebrospinal fluid (CSF), central nervous system (CNS), neurological disorders, interventional radiology, neurosurgery, neuroprotection

## Abstract

Central nervous system (CNS) injury or disease states are often difficult to treat due to the closed system of the dura mater/blood-brain barrier and the bony skull and vertebrae. The closed system results in at least partial containment of any pro-inflammatory molecules, pathogens, or toxic byproducts in the case of brain or spinal cord lesions, which can result in a destructive feedback loop. Cervical-approach access techniques (lateral C1-C2, suboccipital and lateral atlanto-occipital space punctures) are less-common methods of cerebrospinal fluid (CSF) sampling due to the relative ease and safety of lumbar spinal taps. However, with improved image-guidance, these cervical-level CSF access points are still useful when there are certain contraindications and difficulties when attempting to sample the CSF via the typical lumbar spinal approach. With the advent of microcatheters and minimally invasive techniques, combined with body fluid filtration technology, the question arises: could dual microcatheters be introduced for inflow and outflow of purified or artificial CSF to break the destructive feedback loop and thus diminish CNS damage?. We hypothesize that intrathecal spinal catheters could be placed in 2 positions (e.g., via a cervical route and the typical lumbar spinal route) to allow for both an input and output to more effectively filter or “flush” the CSF. This could have broad implications in the treatment of strokes, traumatic brain or spinal cord injury, infections, autoimmune diseases, and even malignancies within the CNS-in short, any disease with abnormalities detectable in the CSF.

## 1 Introduction/background

### 1.1 The problem treating central nervous system lesions

The central nervous system (CNS) comprises the brain and spinal cord. The CNS has a mechanical protective covering from the skull and vertebrae and is isolated biochemically through the blood-brain barrier. While these protective mechanisms have undoubtedly provided evolutionary advantages, such protective mechanisms also serve as a barrier to medical interventions, be they medical in getting pharmaceuticals across the blood-brain barrier, or surgical in getting access through a bony covering. Nevertheless, procedures exist to circumvent the protective structures. For example, lumbar puncture (also known as a “spinal tap”) is a commonly used method to assess the CSF given the limitations in medical imaging.

Cerebrospinal fluid (CSF) serves as both a mechanical and biochemical dynamic buffer to the CNS. As it bathes the CNS, the CSF serves as a unique point of medical intervention. Therapeutics like intrathecal chemotherapy have already exploited the CSF by allowing for the direct injection of cancer treatment into the CSF bathing the afflicted organ. In this paper, we propose the use of “CSF flushing” as a method of treating CNS pathology. We propose a practitioner could perform a cervical approach (lateral C1-C2, sub-occipital or lateral atlanto-occipital space) puncture and drain placement as well as a lumbar puncture and drain placement to simultaneously remove existing CSF and replace it with artificial CSF allowing both input and output points instead of only a single access point as is currently practiced. An intracranial approach (endoventricular or subarachnoid) could alternatively be used as an access point in combination with a cervical and/or lumbar approach if clinically warranted, though such would require traversing the cranial vault. We posit that this CSF flushing method could improve the efficacy of CSF-directed interventions, reducing the severity and length of CNS illness involving CSF, increasing the effectiveness of therapeutic hypothermia, or enabling more controlled stem cell gradients as a few possibilities.

### 1.2 CSF physiology

The physiology of CSF and its anatomical flow is controversial in the literature. Below we describe the traditional conception of CSF flow and contextualize it with new research.

**Figure 1:**
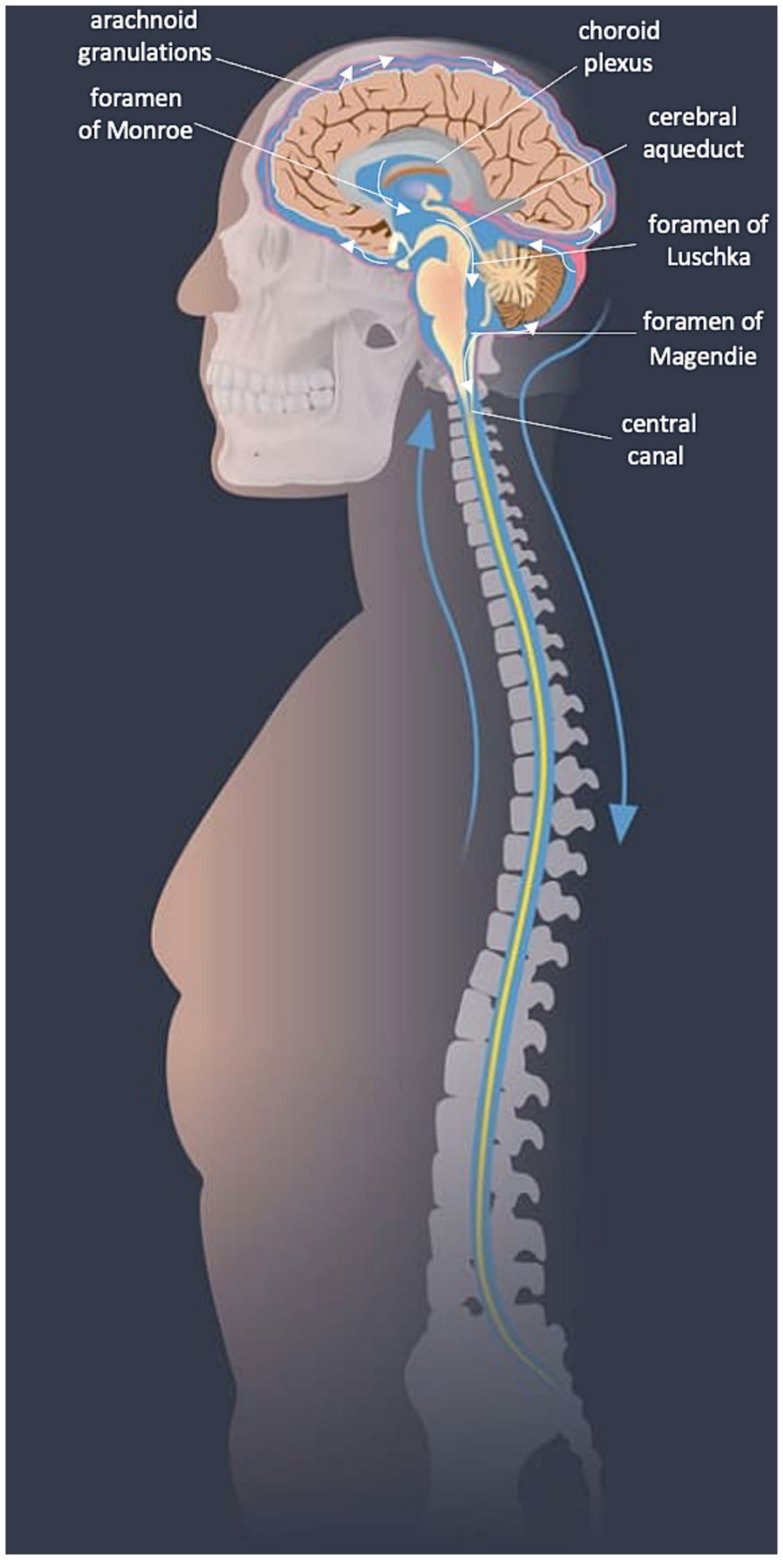
CSF flow diagram. Image modified from SpineUniverse.com.

**Figure 2:**
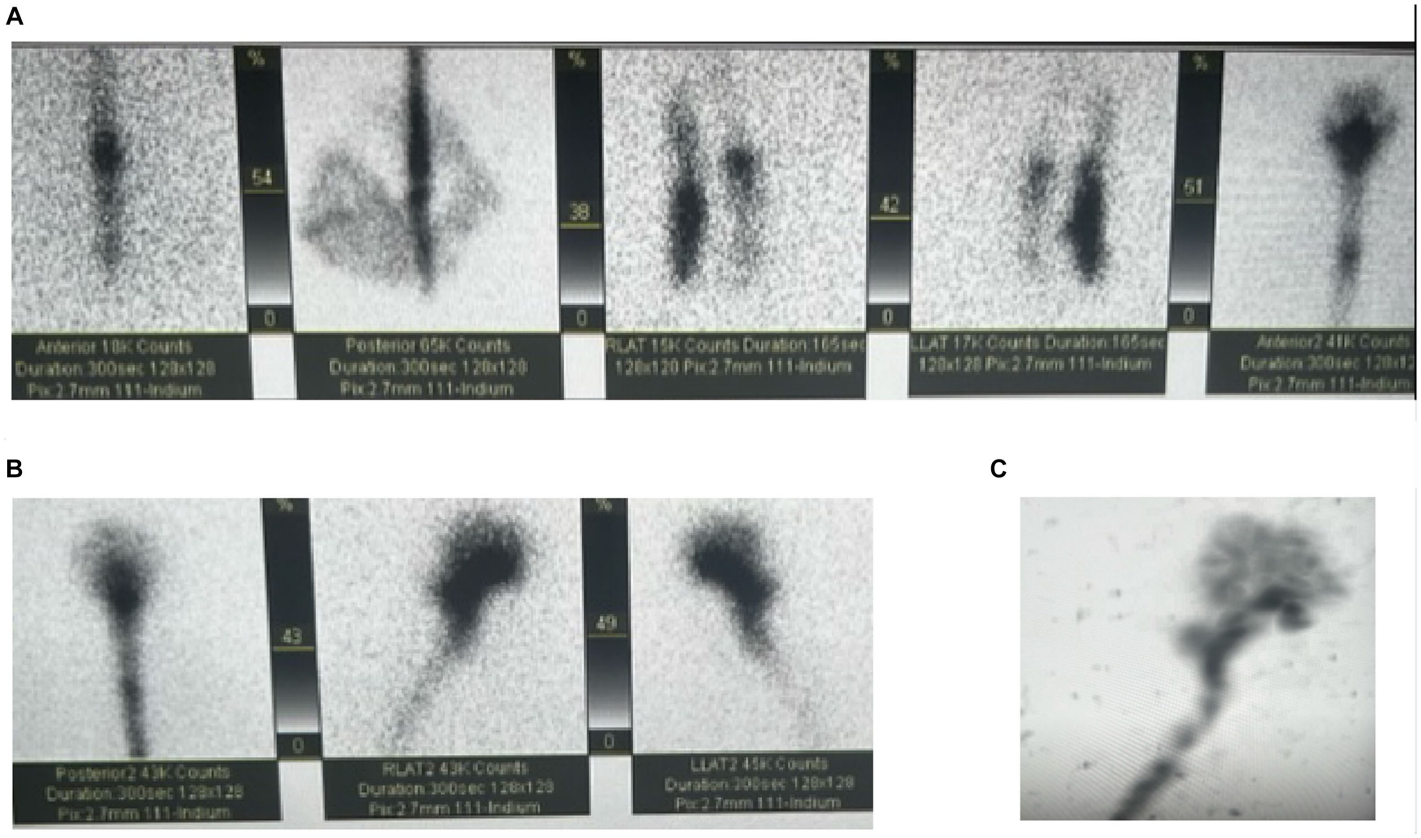
Cisternogram for the evaluation of NPH with intrathecal injection of Indium at the L2-L3 vertebral level. **(A,B)** Demonstrates cranial migration of radiotracer from the injection site. **(C)** Is a maximum intensity projection showing the full flow of the CSF.

The majority of CSF is secreted in a pulsatile manner from tufts of fenestrated capillaries in the lateral ventricles known as “choroid plexuses.” Some CSF is also produced from the *tela choroidea* in the third and fourth ventricles and from the interstitial compartment. Circulation of CSF is primarily rostro-caudal, first draining through the interventricular foramina (of Monro) to the third ventricle, and then to the fourth ventricle through the cerebral aqueduct (of Sylvius). The CSF leaves the fourth ventricle via the median aperture (foramen of Magendie) or lateral apertures (of Luschka) and enters the subarachnoid space or continues to flow inferiorly in the central canal. In the subarachnoid space, CSF diffuses to absorption sites (Sakka et al., 2011) (see Figures 1, 2).

The vast majority of CSF is absorbed into the cranial venous sinuses and epidural venous plexus of the spinal subarachnoid space through arachnoid granulations, which are endothelium-lined protrusions from the venous sinuses into the subarachnoid space. Absorption is facilitated by a 3 to 5 mmHg pressure gradient. There is also a smaller role for CSF absorption by cranial and spinal nerve sheaths, the ependyma, and extracellular fluid (Sakka et al., 2011).

Adults generally secrete anywhere from 400 to 600 mL CSF daily and renew CSF four to five times per 24 h. However, the production rate may vary depending on a person’s demographics, disease state, water consumption, and time of day. At nighttime, as much as a fourfold increase in CSF production has been reported compared to daytime, and even higher than 20 L per day in studies with heavy water intake in humans and animals (Eide et al., 2021). At any given time, adults have approximately 179 mL of CSF in the cranium and 84 mL in the spinal axis for an average total CSF volume of 250 mL or more (Chazen et al., 2017). Adults’ CSF pressure ranges between 10 to 15 mmHg. Increased intracranial pressure (ICP) results in decreased choroidal secretion of CSF (Sakka et al., 2011).

There is some ability for anatomical adaptation to increase CSF. Nevertheless, infusion of saline into the subarachnoid space increases ICP. One review suggested a tenfold increase in ICP following an infusion of 26 mL in adults, 20 mL in a small child, and 8 mL in a neonate, though these values were anecdotally reported with no particular disclosure of the study design, sample size, pressure variability, or how ICP was measured (Sakka et al., 2011). The lumbar infusion test is one important clinical application of CSF infusion. In this test mock CSF is injected in the lumbar region with simultaneous measurement of ICP, with a goal to stress test the ability to reabsorb CSF. Normal pressure hydrocephalus (NPH) is diagnosed if the ICP rises quickly, indicating a reduced capacity to absorb additional fluid (Katzman and Hussey, 1970; Ryding et al., 2018).

New research has shown that the traditional understanding of CSF physiology as described above is likely an over-simplification. For example, the classical theory of CSF flow predicts that a choroid plexectomy should provide relief in patients with hydrocephalus due to decreased CSF production (Brinker et al., 2014). However, a study found that two-thirds of children undergoing this procedure still required shunting due to recurrent hydrocephalus (Lapras et al., 1988). Another study showed that choroid plexectomy in monkeys did not affect the biochemical composition of CSF, indicating a lesser role of the choroid plexus in CSF production and flow in the primate ventricular system (Milhorat et al., 1971). In addition, new studies using MRI techniques have shown that CSF secretion and turnover rates may be up to 40 times as high as traditionally thought (Eide et al., 2021).

Most recently our understanding of CSF physiology has evolved with the discovery of the glymphatic system. In this model, there is a homeostatic relationship between brain vasculature fluid, CSF, and interstitial fluid (ISF). The homeostatic relationships are maintained by three distinct entities: the brain blood barrier (BBB), the perivascular spaces (PVS), and the astrocytic endfeet of the glia limitans (Klostranec et al., 2021).

Anatomically, arteries and veins of the subarachnoid space penetrate the brain parenchyma with adjacent channels known as paravascular spaces that are filled with CSF. The BBB facilitates the osmotic movement of fluid to the paravascular space by allowing for the unidirectional efflux of ions to the paravascular spaces, which is followed by the subsequent migration of water down its osmotic gradient. The paravascular spaces include the perivascular spaces (also known as the Virchow–Robin spaces), which lie between the smooth muscle cells of the penetrating arterial vasculature and the pia mater, and the paravascular spaces that lie between the pia mater and the glia limitans. In the paravascular spaces, the CSF can move to the ISF through aquaporin 4 (AQP4) channels and spaces between the astrocytic endfeet of the glia limitans, or to the venous vasculature (Klostranec et al., 2021).

Convective flow through the glymphatic system is generated by arterial pulsation and respiratory variation. CSF flow oscillations can differ significantly from person to person, and even within a given individual as CSF flow reverts from caudal to cranial during diastole (Klostranec et al., 2021; Vijayakrishnan Nair et al., 2022). The dynamic and bidirectional nature of CSF flow is one explanation for why the protein content of ventricular CSF is different from that obtained from the lumbar region (Puy et al., 2016; Rostgaard et al., 2023). Hence, the location of CSF sampling is important and cannot be assumed to be identical for different locations of CSF fluid.

Of import, studies suggest that with aging the effectiveness of the glymphatic system is reduced as arteries harden and have less pulsative properties, the efficacy of AQP4 channels decreases, and the production of CSF decreases. Low flow throughout the glymphatic system allows for proteinaceous buildup, which can be seen in neurodegenerative diseases like Alzheimer disease (Puy et al., 2016; Klostranec et al., 2021).

In Alzheimer’s disease, there is an alteration in the homeostatic mechanism of the amyloid-β (Aβ) pathway. Aβ peptide is produced by the proteolysis of amyloid precursor protein (APP), which is expressed at high levels in the brain (Knopman et al., 2021). In Alzheimer’s disease there is an accumulation of Aβ, which initially occurs in the cerebral neocortex where metabolic rates are highest, and then spreads to the allocortex and midbrain, and then finally in the late clinical stages of the disease to the cerebellum and brain stem. Accumulation of Aβ is the precursor to the formation of tau neurofibrillary tangles and neuronal and synaptic loss, which are other hallmarks of Alzheimer’s disease (Hampel et al., 2021). Therefore, Aβ accumulation precedes neurodegeneration and cognitive decline.

In an average healthy adult, the rate of Aβ production and clearance are roughly balanced at 7.6% per hour and 8.3% per hour, respectively (Bateman et al., 2006). Therefore, a small increase of Aβ production or a small reduction in clearance can be enough to upset homeostasis and cause the aggregation of Aβ. For example, the apolipoprotein E ε4 allele upregulates APP transcription and subsequently increases Aβ production and is, therefore, a risk factor for early-onset Alzheimer’s disease. On the clearance side, a decrease in function of the glymphatic system with aging causes an aggregation of Aβ, thereby upsetting the natural homeostasis of Aβ production and clearance (Hampel et al., 2021; Knopman et al., 2021). As Aβ aggregation is the first manifestation of Alzheimer’s disease, early therapeutic intervention that promotes increased clearance of the Aβ peptide has the potential stop the progression of Alzheimer’s disease, before neuronal and synaptic loss occurs.

### 1.3 Techniques and approaches to accessing CSF

The most common way to sample the CSF is through a lumbar puncture, colloquially known as a “spinal tap.” To avoid any spinal cord injury, a needle is placed between vertebral bodies below the level of the filum terminale of the spinal cord. While done much less frequently, CSF can be sampled via a lateral atlanto-occipital space puncture (also called lateral cisterna magna puncture), a lateral C1-C2 approach, or even sub-occipital puncture (though with the risk of hitting the brainstem and associated complications described below). A more permanent solution to sample or divert CSF is to do a ventriculostomy where an external ventricular drain is placed through the skull and cerebral cortex to within the cerebral ventricles.

#### 1.3.1 Lumbar puncture

Lumbar puncture (LP) is performed to sample CSF (most commonly), measure intracranial pressure (ICP), lower ICP by draining CSF, and inject a small volume of therapeutics. The patient is positioned in the lateral recumbent position or the sitting position, where the patient sits on the edge of a bed and arches their back. LP technique is generally performed with an atraumatic or small diameter needle in the L3/L4 or L4/L5 interspace to avoid hitting the spinal cord as it terminates around L1/L2 (Wright et al., 2012).

Contraindications of LP include infection at or above the LP site, thrombocytopenia, low international normalized ratio (INR), developmental abnormalities like myelomeningocele, and increased ICP. Concern for elevated ICP should exist when the patient is over age 60, immunocompromised, has a history of CNS disease, recent seizure, reduced consciousness, papilloedema, or an abnormal neurological examination. In such cases, a CT head should be performed to rule out elevated ICP (Wright et al., 2012).

Common complications include discomfort at the needle entry site and post-LP headache. Post-LP headache is more common with traumatic needles (26% of patients) versus atraumatic needles (9% of patients). More rare complications include infection (<0.01%), spinal hematoma (<0.01%), and persistent CSF leak. CNS herniation can also occur in cases of elevated ICP, as the removal of CSF allows the raised pressure compartment above the LP to move along the pressure gradient and consequently move CNS tissue (Wright et al., 2012).

Overall LP is a commonly performed procedure that is generally well tolerated.

#### 1.3.2 Sub-occipital and lateral C1/C2 puncture

**Figure 3:**
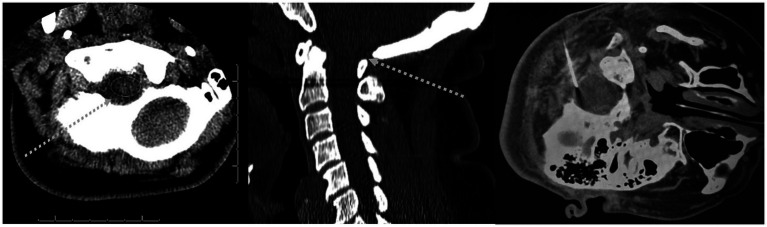
Suboccipital approach. (Left and middle panels): Dashed arrow indicates off-plane needle trajectory on a typical diagnostic CT. (Right panel): CT-guided sub-occipital needle placement.

The sub-occipital approach was described in 1919 (Wegeforth et al., 1919; Ayer, 1920). The patient is placed on their side with their neck flexed and a needle is placed midline just above the atlas. The needle is then aimed towards the superior aspect of the auditory meatus and advanced through the skin into the cisterna magna (Ayer, 1920). The midline sub-occipital approach fell out of favor because of reports of subarachnoid hemorrhage (SAH) and direct puncture of brain tissue. Most reported incidents occur secondary to anatomic variation where a patient may have large midline PICA branches, small posterior fossa, or inferior displacements of brainstem and cerebellar tonsils (Keane, 1973). However, with CT-guidance, this can still be performed with relative safety. Figure 3 illustrates the suboccipital approach.

The lateral C1/C2 puncture was created as a safer alternative to the midline suboccipital approach as there is less vasculature and brain tissue in the C1/C2 interspace than in the posterior fossa. This approach is widely taught at neuroradiology programs across the US, and generally reserved for situations when LP or MRI myelography is contraindicated (Daniels et al., 2019).

**Figure 4:**
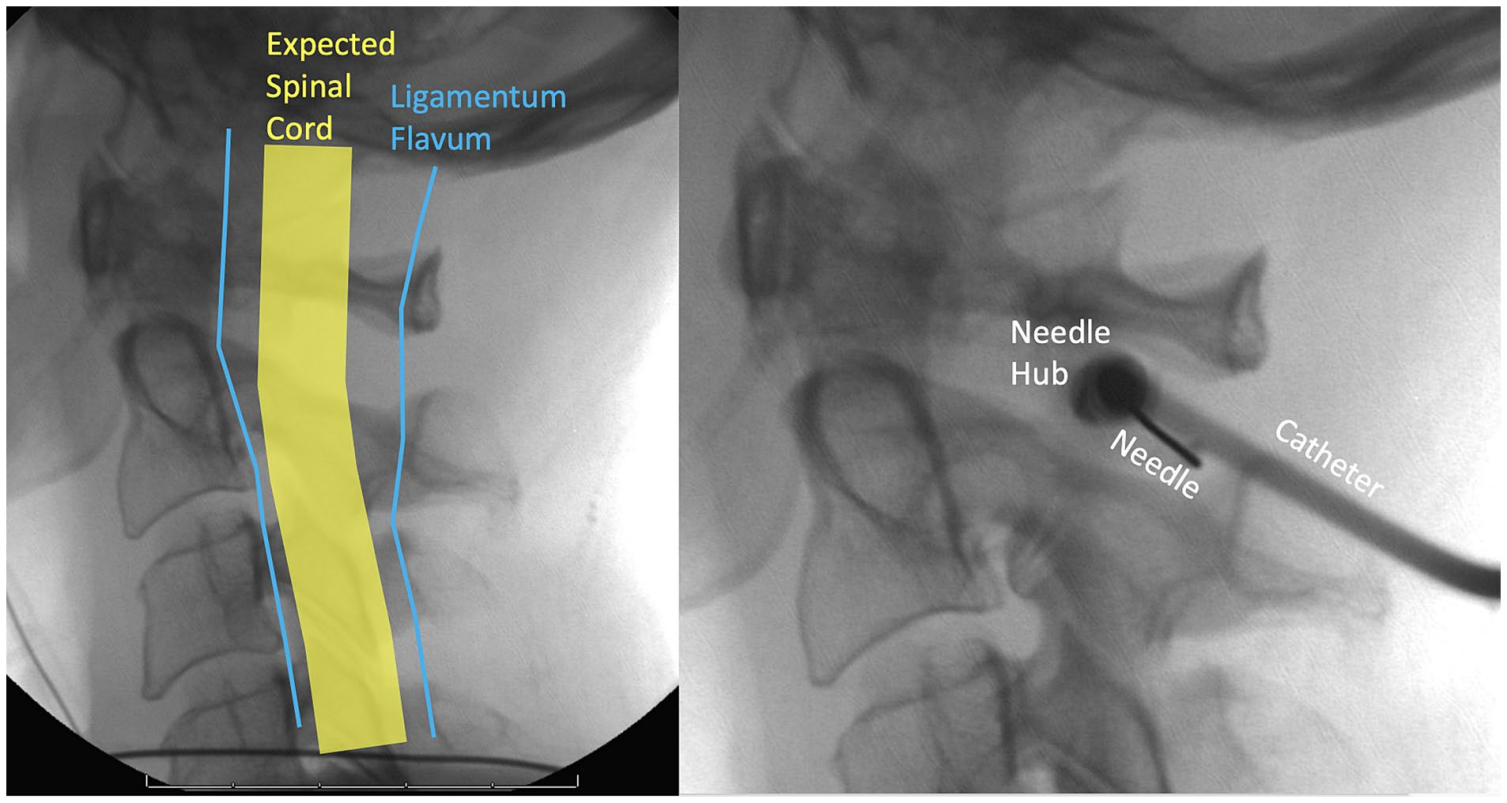
Fluoroscopic lateral C1-C2 planning and needle entry. To widen the space between the spinal cord and the ligamentum flavum the patient should flex their neck anteriorly as much as possible.

The C1/C2 interspace is used because it is difficult to safely perform a puncture below C2 because the spinal cord widens at lower vertebral levels. The patient is supine or prone and the technique is done under fluoroscopy or CT guidance. A needle is introduced into the posterior third of the spinal canal at the C1/C2 interspace from a lateral approach, 2–5 mm anterior to the spinolaminar line and 4–6 mm inferior to the C1 arch (Yousem and Gujar, 2009; Daniels et al., 2019). Figure 4 illustrates the lateral C1-C2 planning and puncture.

Major complications of lateral C1/C2 puncture include vertebral artery puncture, PICA laceration, and neurological injury (Yousem and Gujar, 2009; Daniels et al., 2019). Research has shown that there is large anatomic variation in the course of the vertebral arteries in the C1-C2 region and that approximately 1% of patients have PICA’s that are susceptible to injury in a C1-C2 puncture (Cacciola et al., 2004; Brinjikji et al., 2009). Neurological injury can also occur due to puncture of brain tissue. Other complications of lateral C1/C2 puncture are similar to an LP. In a mail survey of 220 neuroradiologists serious complications of C1-C2 puncture was estimated at around 0.05%, but this study was limited by response bias and the complication rate may be 20 times higher compared to an LP (Robertson and Smith, 1990; Daniels et al., 2019). Overall, the C1/C2 puncture is effective and safe, but only when done under image guidance like fluoroscopy or CT (Yousem and Gujar, 2009; Daniels et al., 2019).

**Figure 5:**
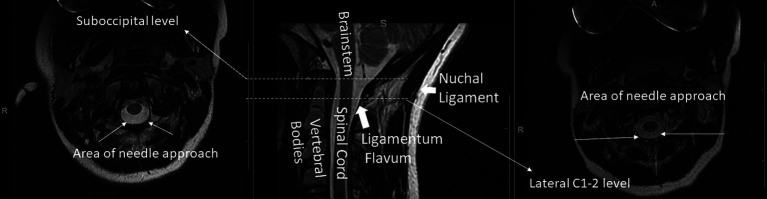
Axial T2 at the suboccipital (left) and lateral C1-2 (right) levels and corresponding mid-sagittal T2-weighted (middle) cervical spine MRI with a few key structures labeled.

Figure 5 is an anatomic overview showing where the suboccipital puncture and the lateral C1-C2 puncture is performed.

#### 1.3.3 Lateral atlanto-occipital space puncture

The lateral atlanto-occipital space puncture was developed in the 1990s as a safer alternative to the sub-occipital and lateral C1-C2 punctures. In this procedure the patient is placed supine with the head and neck straight, and a 20-gauge-needle is advanced perpendicular to the neck 1 cm inferior and 1 cm posterior to the highest part of the mastoid. An advantage of this technique over the suboccipital or lateral C1/C2 puncture is that the vertebral artery less at risk of injury (the vertebral artery runs in the intervertebral foramen of the C1/C2 intervertebral space). One 2017 retrospective case series of 1,008 lateral atlanto-occipital space punctures performed on 667 patients found that 98.3% of punctures were successful (Gong et al., 2018). No serious complications were found and 5% of patients experienced transient complications that included ipsilateral ear pain (2.25%), increase in blood pressure (4.80%), and intracranial hypotension (0.15%) (Gong et al., 2018). This case series concluded that the lateral atlanto-occipital approach is a safe and easy way to access the subarachnoid space and is safe to do without fluoroscopy or CT guidance (Gong et al., 2018).

#### 1.3.4 Ventricular shunt or endoventricular drain

A ventricular shunt or endoventricular drain is yet another way to access CSF, where a neurosurgeon inserts a catheter into a lateral ventricle through a burr hole at a desired entry point in the skull. Complications of ventricular shunts include infection, intraventricular hemorrhage, malposition, obstruction, seizures, subdural hemorrhage (Merkler et al., 2017). It is estimated that approximately one-third of patients will experience at least one complication and may require revision surgery for long-term shunts. Hemorrhage occurs in approximately 7% of patients, and severe hemorrhage in approximately 0.8% of patients (Bauer et al., 2011). Removal of a ventricular shunt is infrequent, but possible. In a retrospective study of 661 patients with hydrocephalus treated over a 10 years period, 10 patients underwent an attempt at shunt removal with 7 removals successful (Al-Holou et al., 2010).

#### 1.3.5 Lumbar drain

A lumbar drain (LD) may be used for temporary CSF flow diversion in cases of abnormal CSF flow or increased volume, such as CSF rhinorrhea, pseudomeningocele, and NPH. It is performed similar to a lumbar puncture, first by advancing a needle into the intrathecal sac at the L3/L4 or L4/L5 interspace. Once the ligamentum flavum is punctured and clear fluid flows from the needle, a catheter is advanced over or through the needle into the intrathecal sac. The needle is removed once the catheter is in place (Bakhshi et al., 2020).

Traumatic CSF rhinorrhea is responsible for 90% of all cases of CSF leakage. A LD is commonly used when conservative therapy fails to manage CSF rhinorrhea. A LD works by decompressing intracranial pressure, thus decreasing CSF flow through the defect to promote healing and brain relaxation. LDs may be more common than it is helpful in the management of CSF rhinorrhea. In one retrospective study of patients with recurrent CSF rhinorrhea, 32 were treated conservatively without LD and 29 were treated with LD after conservative therapy failed (Bakhshi et al., 2020). Their results showed no difference between the groups in recurrence of CSF leakage or incidence of meningitis; however, hospitalization was longer by 8 days in the LD group on average. Therefore, they concluded that LD is not an appropriate technique for management of CSF leakage. However, its use as an adjunct in addition to conservative therapy should still be investigated.

Another retrospective study of 254 patients with NPH who received LD trial revealed that 30% of patients had improvement in gait, cognitive decline, or urinary incontinence following the LD intervention (El Ahmadieh et al., 2019). Complications included meningitis, lumbar epidural abscess, CSF leak at insertion site, transient lower extremity numbness, slurred speech, refractory headaches, and hyponatremia. A history of stroke was associated with poorer outcomes, while disproportionate supratentorial subarachnoid enlargement predicted better outcomes (El Ahmadieh et al., 2019).

**Figure 6:**
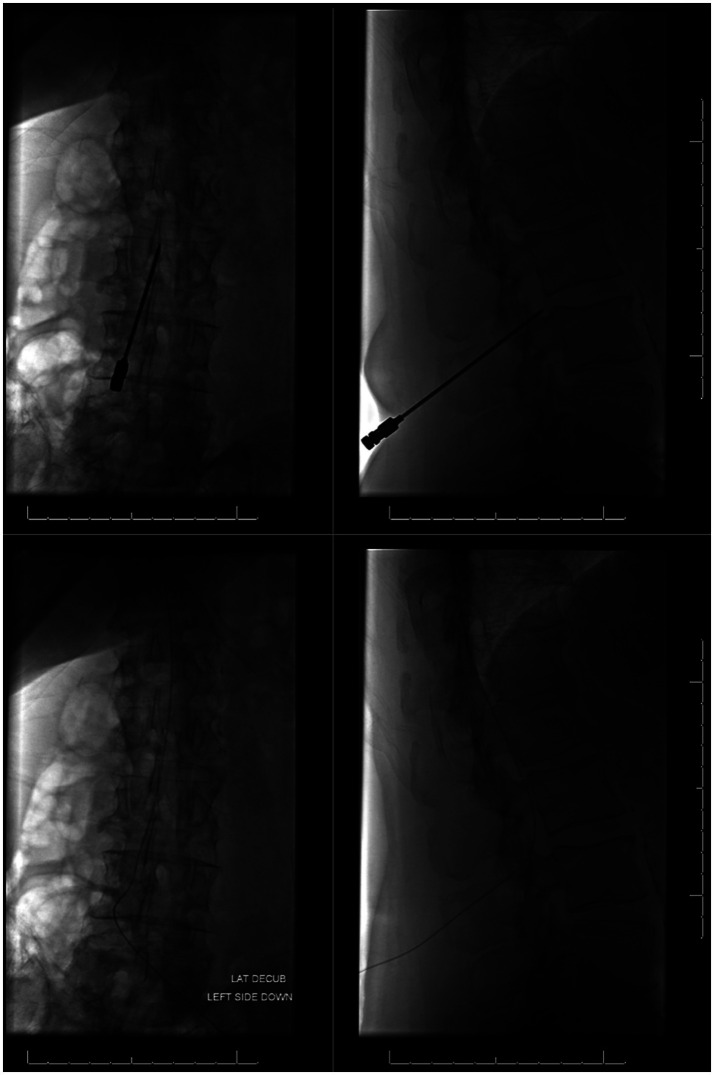
Placement and final positioning of a lumbar drain using fluoroscopic guidance. Images on left illustrate a frontal view and images on right show a lateral view.

Figure 6 illustrates placement and final position of a lumbar drain.

#### 1.3.6 Dual lumen microcatheter

Using a dual-lumen microcatheter for CSF exchange would allow for the simultaneous infusion and extraction of fluid into/out of the CSF space.

**Figure 7:**
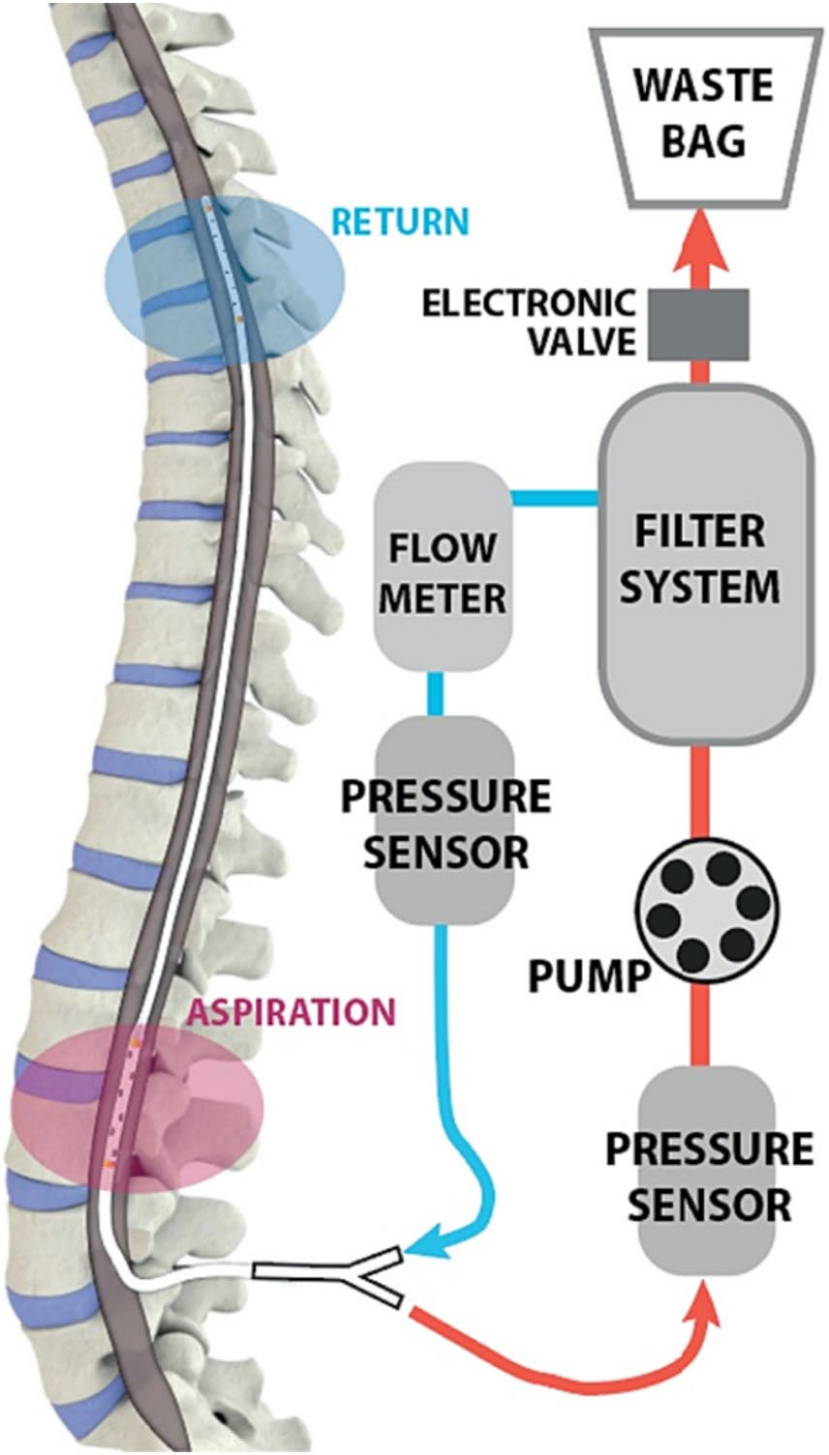
Diagram of Neurapheresis system, borrowed from Blackburn et al. (2019). A dual-lumen microcatheter is introduced in the lumbar region. The catheter is connected to a pump and an external filter system. CSF is drawn into the filter, which removes waste products. Remaining CSF is returned to the patient.

Human trials of intrathecal dual-lumen catheters in the setting of aneurysmal subarachnoid hemorrhage have already begun. As red blood cells from a SAH break down, hemoglobin and proinflammatory cytokines are released that are associated with complications like vasospasm and hydrocephalus (Blackburn et al., 2019). Although lumbar drains have previously been used to remove blood products from patients with subarachnoid hemorrhage, those are limited by the rate of CSF production. In contrast a CSF exchange system that filters blood from the CSF and returns it to the patient is not limited by rate constraints. In 2019, one prospective study completed the first human trials of the Neurapheresis system, a pump system employing a dual-lumen catheter that simultaneously extracts blood-tinged CSF, filters out blood products, and re-infuses filtered CSF (Blackburn et al., 2019). The dual-lumen catheter in the system is connected to a filtration unit external to the patient that the clinician uses to control the rate of CSF filtration. Figure 7 illustrates the Neurapheresis system, as used in the 2019 study.

Parameters monitored during the trial included system pressures and flow rates. Neurologic status remained stable or improved in 92% of patients. Pump flow ranged between 30–96 mL/h and waste production between 12–15 mL/h. In patients whose CSF was filtered for at least 22 h there was a mean red blood cell reduction of 80%. Of the 13 patients in the trial, none had severe adverse events associated with catheter placement. These results demonstrate safety and feasibility of the Neurapheresis system; however, evaluation of treatment efficacy requires larger sample sizes and longer pump durations (Blackburn et al., 2019).

### 1.4 Optimization and engineering modeling of CSF filtration

There has been research on how to refine current CSF filtration strategies. Due to the complex geometry of the subarachnoid spaces, it is difficult to accurately quantify intrathecal CSF flow rates. Several engineering modeling studies have used computational fluid dynamics (CFD) to model CSF flow. Below some of the foundational studies in the field are summarized.

One early study investigated the effect of a lumbar drain on blood clearance following SAH in a bench-top model of CSF dynamics, based on 3D anatomical data from a healthy 29 years-old volunteer (Tangen et al., 2016). Porcine blood was injected into the model to stimulate a hemorrhagic event, and the efficacy of lumbar drains was assessed for different drainage rates and patient orientations. A smoothed CFD model with idealized geometry was used to computationally estimate blood clearance, and these results were compared to that of the physical model. The results of both the bench-top model and the CFD model showed that performing lumbar drainage in patients in an upright position maximizes fluid diversion from the cranial CSF compartment, thereby enhancing blood clearance (Tangen et al., 2016). Because the trends observed in the physical bench-top model and the computational CFD model were similar, the authors conclude that CFD models are promising tools for studying blood clearance from the CSF.

In another study, the intrathecal CSF dynamics of the Neurapheresis system were investigated in post-subarachnoid hemorrhage patients using a subject-specific CFD model with idealized geometry (Khani et al., 2019). This model simulated various Neurapheresis flow settings and compared them to a lumbar drain simulation with a typical CSF flow rate of 0.2 mL/min. Results showed that at a maximum flow rate of 2.0 mL/min, the Neurapheresis system had a twofold higher average steady streaming CSF velocity compared to a lumbar drain. This suggests that the Neurapheresis system can be beneficial for improving CSF dynamics in post-SAH patients, potentially limiting adverse events and shortening hospital stays (Khani et al., 2019). The major limitations of this CFD model are that it did not consider intracranial CSF flow, lacked some fine anatomical structures of the CNS, and did not incorporate a multiphase fluid mixture of blood and CSF.

In another study, authors built a subject-specific multiphase CFD model of CSF solute transport based on MRI measurements to investigate the effect of the Neurapheresis system on hemorrhage filtration, when compared to a lumbar drain (Khani et al., 2020). The results of the CFD system were verified by using an identically constructed *in-vitro* model. The authors found that the aspiration and return rate for filtrate was 2.0 and 1.8 mL/min for the Neurapheresis system, versus 0.2 mL/min for lumbar drain. The maximum increase in steady-streaming CSF velocity was observed in the intrathecal region between the aspiration and return ports (Khani et al., 2020). The advantage of the CFD model used in this study was that it was more anatomically accurate than prior models and incorporated a multiphase component of CSF flow.

In a preprint another set of authors showed that CFD modeling with T2-weighted magnetic resonance imaging can be used to accurately visualize CSF dynamics. Based on a single, healthy 42 years-old Caucasian male, researchers developed a computational framework via 3D Slicer software and COMSOL Multiphysics^®^ v6.0 that integrates fine aspects of CSF flow through patient-specific cranial CSF space, ventricular system, and periarterial spaces (Misiulis et al., 2023). This model was able to maintain a constant net CSF flow, while also providing insights into pressure and momentum distribution. Unlike previous computer engineering simulations that simplified the geometry of the brain parenchyma and perivascular spaces, this model incorporates a high level of anatomical detail for CSF flow patterns, and is therefore applicable to multiple clinical scenarios, such as SAH, cerebral ischemia, multiple sclerosis, traumatic brain injury (TBI), and hydrocephalus (Misiulis et al., 2023).

Overall, CSF flow modeling is a rapidly evolving field that increasingly recognizes the complexity of CSF dynamics and seeks to incorporate this complexity into the creation of more accurate computational models. These models will prove useful to create new and fine-tune existing CSF filtration strategies, each tailored to a patient’s specific clinical presentation and anatomy.

### 1.5 Changes in CSF associated with CNS pathology

Waste products from metabolic activity in CNS tissue diffuse into the CSF and then travel to the bloodstream for removal. In many degenerative/inflammatory CNS disorders the composition of CSF changes with pathology. Table 1 illustrates some common CNS pathologic states and their respective CSF changes.

**Table 1:** CSF abnormalities in neurological disorders [see references (Robertson and Smith, 1990; Deisenhammer et al., 2019; Robey and Panegyres, 2019; León-Lozano et al., 2020) for details].

Degenerative	
Alzheimer disease	Elevated CSF Aβ_42_, decreased Csf T-tau, decreased CSF P-tau
	Elevated CSF Aβ_42_/Aβ_40_ ratio
Frontotemporal dementia	Reduced CSF P-tau_181_/T-tau
Parkinson’s disease	Nonspecific markers: NfL, α-synuclein, Aβ42, T-tau or P-tau
Motor neuron disease	Elevated NfL and pNFH; uncertain specificity

Inflammatory/infectious	
Multiple sclerosis	Oligoclonal bands, lymphocytosis, elevated IgG
Prion disease	Elevated 14-3-3 protein
Microbial encephalopathy	Virion particles, bacteria, fungi, parasites

Traumatic/vascular/hemorrhagic	
Hypoxic-ischemic encephalopathy	Elevated NSE
Subarachnoid hemorrhage	Extravasated hemoglobin

Because the CSF takes on the pathologic state of the CNS pathology in all the disease states described in Table 1, removing this pathologic CSF and replacing it with non-pathologic CSF (or artificial CSF substance, hereafter termed “CSF flushing”) may lead to better clinical outcomes. Indeed, manipulation of the CSF provides a unique opportunity for therapeutic intervention.

## 2 The hypothesis/theory

### 2.1 CSF flushing decreases CNS illness severity and duration

The main hypothesis is that CSF flushing using 2 (or more) CSF access points could reduce the concentration of disease-causing molecules and organisms, in turn decreasing the severity and/or duration of CNS-lesions involving the CSF. Additionally, CSF flushing with cool saline/artificial CSF could augment systemic therapeutic cooling.

### 2.2 Evaluation of the hypothesis/theory

Cerebrospinal fluid lavage is performed using neutral physiological fluids, such as saline (0.9%), Ringer’s lactate and artificial CSF (aCSF). aCSF simulates CSF in the main chemical indexes including sodium (Na^+^), potassium (K^+^), magnesium (Mg^2+^), calcium (Ca^2+^), chlorine (Cl^−^), phosphorous (P), bicarbonate (HCO_3_^−^), glucose, pH and osmotic pressure thereby limiting potential side effects during CSF exchange. Although aCSF is the preferred solvent for CSF flushing, lactated Ringer’s solution and normal saline are alternative crystalloids that are commonly used for neurosurgical irrigation (Kazim et al., 2010; Mori et al., 2013; Zheng et al., 2020). Table 2 shows the benefits, limitations and potential risks for substances used in CSF exchange.

**Table 2:** Irrigation for CSF Lavage [see references (Kazim et al., 2010; Mori et al., 2013; Zheng et al., 2020) for details].

Artificial CSF (aCSF)	Most similar to CSF (pH, osmolality, ion content)
	Minimizes cerebrovascular permeability and cell damage
	Expensive
Normal saline	Most commonly used, cost effective
	Different pH, ion composition, osmolality compared to CSF
	Higher risk of neurotoxicity, cerebral edema, fever, headache, seizure
Lactated Ringer’s	Electrolyte concentrations (Na^+^, K^+^, Cl^−^, lactate, Mg^2+^) are different from CSF
	Safe alternative to aCSF
	Minimal risks, may cause cerebral edema, symptomatic hyperkalemia

#### 2.2.1 CSF exchange improves neurological outcomes in animal models

CSF exchange therapy aims to improve the CNS milieu by removing pathologic CSF and replacing it with artificial CSF enhanced with protective agents. One study used an intracerebroventricular CSF exchange procedure in mice that had been induced with chronic experimental autoimmune encephalomyelitis (EAE), a pathology like multiple sclerosis (Deisenhammer et al., 2019). Researchers found that the removal of endogenous CSF and the infusion of artificial CSF enhanced with human mesenchymal stem cell secretions (MSCs) delayed the onset of EAE and reduced symptoms (Valitsky et al., 2019). Importantly, infusion of artificial CSF that had been enhanced with MSCs without the removal of endogenous CSF only delayed the onset of EAE. Hence, there is evidence that removing pathologic CSF is important to ameliorate the clinical sequelae of degenerative/inflammatory CNS disorders (Valitsky et al., 2019).

Other scientists used a similar experimental approach to the research study above but used mice with Alzheimer disease (Benhamron et al., 2020). Alzheimer disease has been linked to decreased CSF production with associated increased resistance to CSF outflow. This CSF flow abnormality reduces the efficiency of the body to remove CSF wastes like beta-amyloid and Tau protein. This study found that CSF exchange therapy improved cognition, increased neuron counts, reduced astrocytic burden, and increased cell proliferation and neurogenesis in a dose responsive manner where increased frequency of CSF exchange therapy created more pronounced effects (Benhamron et al., 2020).

A recent preclinical model that uses CSF filtering extracted CSF from 11 amyotrophic lateral sclerosis (ALS) patients (Venkatraman et al., 2022). Some of the extracted CSF was then filtered with 100 kD molecular weight cut-off filters to remove key proteins known to be involved in ALS. Thirty-six mice were divided up into three groups of 12, and each group then received cisterna magna injections of unfiltered ALS CSF, filtered ALS CSF, or saline. The authors found that unfiltered ALS CSF injected into the cisterna magna induced motor deficits in mice, while such deficits were not induced in mice that received filtered ALS CSF or saline. Hence the authors demonstrate that it is possible to filter the ALS-causing protein from the CSF of ALS patients, and that filtering ALS CSF can potentially prevent disease occurrence.

Lastly, CSF flushing has been shown to be effective in improving outcomes in those with spinal cord ischemia. One study found that intrathecal infusion of oxygenated-artificial CSF (OA-CSF) infusion mitigates spinal cord ischemic injury in a rabbit model involving infrarenal aortic balloon occlusion (Kanda et al., 2016). The rabbits receiving OA-CSF infusion had significantly increased CSF partial pressure of oxygen, increased preservation of anterior horn cells, and decreased neuronal degeneration compared to rabbits that did not receive OA-CSF. However, the infusion group did experience a rise in intracranial pressure, thus, researchers recommend the use of an additional intrathecal catheter or use of a single, double-lumen catheter for purposes of CSF drainage and regulating intracranial pressure. They concluded that OA-CSF infusion may protect against sequelae associated with ischemic injury (Kanda et al., 2016).

Overall, existing research indicates that CSF flushing shows promise for several CNS disorders.

#### 2.2.2 Human CSF exchange has been trialed before

A case study describes a patient that had been inadvertently given an mezlocillin overdose and subsequently developed recalcitrant serial epileptic seizures (Kristof et al., 1998). To prevent spread of the mezlocillin from the ventricles into the subarachnoid space, CSF exchange was performed. CSF drainage was accomplished through a ventricular catheter and artificial CSF was infused into the lumbar subarachnoid space. The patient recovered to their baseline pre-mezlocillin and suffered no adverse complications from the procedure (Kristof et al., 1998).

Likewise, another patient was successfully treated from an intrathecal methotrexate overdose by flushing normal saline through a ventricular cannula while simultaneously removing CSF with an LP. The patient in this case recovered fully within a week following the overdose, and CT revealed no structural abnormalities that resulted from the procedure (Spiegel et al., 1984).

Additionally, CSF exchange can be implemented to potentially treat neurodegenerative diseases. One investigation tested liquorpheresis (CSF filtration) as a method to ameliorate Guillain–Barré syndrome (GBS) symptoms in six patients (Wollinsky et al., 1991). Researchers performed a LP and used a closed system to remove CSF, filter the CSF through two 0.2 μm filters, and then re-infuse the filtered CSF. Although the investigators could not understand exactly what changed in the CSF composition to improve symptoms, the results showed that GBS-related symptoms abated rapidly following multiple procedures (Wollinsky et al., 1991).

CSF exchange has also been proved effective in improving patients’ outcomes from infection. As an example, two reported cases of pyogenic ventriculitis have been treated intraventricularly with antibiotics through a dual lumen microcatheter system (Rezai Jahromi et al., 2021). One lumen delivered an antibiotic solution while the other lumen drained purulent fluid and monitored intracranial pressure. Both patients fully recovered without any postoperative complication.

#### 2.2.3 CSF exchange with pseudodelivery of drugs

The therapeutic effects of CSF exchange for treating neurodegenerative diseases could be enhanced when used in conjunction with intrathecal (IT) pseudodelivery of drugs. With image-guidance and microcatheters, drug delivery systems (DDS) containing either enzymes, antibodies, or transport proteins can be used to remove CSF wastes without the actual delivery of the therapeutics contained (hence the name “pseudo”-delivery) (Manuel et al., 2023). This is accomplished in the DDS via nanoporous membranes. Nanoporous membranes allow the influx of small, targeted molecules into the DDS, while preventing the efflux of larger therapeutic molecules, like enzymes or antibodies (Manuel et al., 2023). For IT pseudodelivery to be effective there must be a drug that has selectivity for a target CSF molecule, and a significant size difference between the drug and targeted molecule (Manuel et al., 2023).

There are several examples of therapeutic agents that can be administered via IT pseuodelivery for treating neurodegenerative diseases. Monoclonal antibodies, like aducanumab and lecanemab, can be directed against misfolded proteins such as Aβ, tau protein, and α-synuclein in Alzheimer disease. Anti-TNF agents, which are approved for autoimmune conditions, have also shown promising results. For example, one study found that intraventricular administration of infliximab reduced Aβ plaques and tau phosphorylation in APP/PSL mice (Shi et al., 2011). In another case study, a newly diagnosed patient with Alzheimer’s disease received an infliximab injection via lumbar puncture, which rapidly improved her cognitive symptoms for approximately 30 days before returning to baseline (Shi et al., 2011). Though these studies suggest TNF-α—inhibitors reduce neuroinflammation and improve cognitive deficits, larger controlled trials are needed.

Compared to other routes of therapeutic delivery, IT pseudodelivery preserves non-targeted CSF proteins, acts continuously, and minimizes unwanted effects of systemic medications. While this avenue of drug administration is still in the preclinical stage, it has potential to extend the lives of those who suffer from neurodegenerative diseases.

#### 2.2.4 CSF exchange could be combined with a cooling system to augment therapeutic hypothermia

Patients suffering from ischemia of all causes fundamentally have a mismatch between metabolic supply and demand via decreased oxygen perfusion to metabolically active tissues. Therapeutic hypothermia achieves a lower metabolic demand to match the reduced oxygen supply in ischemia. Therapeutic hypothermia acts on a variety of intracellular pathways to reduce oxygen demand and promote tissue survival, including preservation of ATP through decreased activity of Na/K/ATPas (Storey et al., 2007), activation of pro-survival Bcl-2 (Shuja et al., 2009), and downregulation of TNF receptor and Fas protein expression (Lotocki et al., 2006).

In a 2017 meta-analysis, 41 studies in adults and eight studies in children were examined to assess the use of hypothermia for treatment of TBI. The study found that those who underwent therapeutic cooling had an 18% reduction in mortality and 35% improvement in neurologic outcome compared to those that did not undergo therapeutic cooling (Crompton et al., 2017). However, the meta-analysis also found that therapeutic cooling was associated with adverse outcomes in pediatric patients (Crompton et al., 2017). This led researchers to question whether therapeutic cooling could be used in conjunction with CSF exchange to improve morbidity and mortality of all patients suffering from TBI.

This was investigated in several studies, assessing the possibility of combining intranasal mesenchymal stem cells (MSCs) with the administration of acute hypothermia as a potential treatment for hypoxic-ischemic brain injury. One study found that although each treatment individually provided neuroprotective benefits, combining intranasal MSCs with hypothermia reversed any protective effects (Herz et al., 2018). On the other hand, another study found that hypothermia augments neuroprotective activity of intraventricularly injected MSCs (Park et al., 2015). Although further exploration is needed in this area, we posit that CSF exchange could also be combined with therapeutic cooling for rapid therapeutic relief for patients with TBI and hypoxic–ischemic injury.

## 3 Consequences of the hypothesis and discussion

As has been described, changes in the CSF milieu are common in neurologic disease. Basic science research has provided promising results so far for replacing pathologic CSF with artificial CSF and other chemical and biologically active substances. Infusion of mesenchymal stem cells with artificial CSF has good preliminary evidence. Some potential benefits include symptom amelioration, delaying the onset and/or severity of disease, and reducing CNS inflammation.

In addition, some neurological diseases do not only produce alterations in the chemical milieu of the CSF, but also in mechanical properties through flow alteration. As described earlier, Alzheimer disease is associated with decreased production of CSF and increased resistance to outflow. CSF exchange offers a method to bypass dysfunctional CSF flow that may be present in diseases, and thereby improve clearance of toxins. An additional indication or benefit in the acute setting that CSF exchange may offer is the potential to augment therapeutic hypothermia cooling by allowing for the direct cooling of the CSF.

Of note the manipulation of the CSF milieu for the treatment of Alzheimer disease is controversial. In one double-blind, randomized, placebo-controlled trial, 215 subjects with Alzheimer disease underwent ventriculoperitoneal shunt placement or sham (occluded) shunt placement to see if improved clearance of macromolecules from the CSF milieu would slow the progression of dementia (Silverberg et al., 2008). The primary endpoint was the Mattis dementia rating scale (MDRS), with secondary endpoints of CSF amyloid-beta peptides and CSF abnormally phosphorylated tau protein. The study found that there was no difference between the two groups at 9 months in MDRS scores and biomarkers, and that the MDRS results were not significantly different from the start of the study for either group. However, that study’s conclusion does not conflict with our hypothesis for two reasons. First, the study only looked at removing CSF and not replacing it, so it was not a true CSF flushing mechanism. Second, the authors found that biomarkers significantly decreased in both the experimental and sham groups following ventriculoperitoneal shunt placement. Hence this raises the possibility that the placement of the occluded ventriculoperitoneal shunt in the sham group may have created an inadvertent CSF shunt pathway. In this case comparisons between the two groups would not accurately reflect true differences between intervention and no intervention groups.

Techniques for accessing CSF are many and can be performed safely under image guidance. We posit that CSF exchange could be performed in multiple different ways: (Sakka et al., 2011) a cervical catheter and a lumbar catheter connected to a pump that promotes unidirectional CSF flow to replace pathologic CSF with artificial CSF, (Eide et al., 2021) a ventricular catheter and a cervical drain, or (Chazen et al., 2017) a ventricular catheter and lumbar drain, and (Katzman and Hussey, 1970) a dual-lumen catheter allowing for optional bidirectional flow inserted into any of the above CSF access points. Therapeutic CSF exchange could be on an intermittent basis by interventional radiology or neurosurgery or could be performed continuously with an implanted pump system that a patient could carry around on their own.

Regardless of how the CSF exchange is performed, it is important that protocols begin to be developed for best practices. As CSF exchange as a therapeutic measure is still in its infancy, no protocols currently exist. Future research should explore the creation of such protocols. Developing CSF exchange protocols tailored to specific disease states may be more beneficial than developing one single standardized approach.

Overall CSF exchange offers exciting possibilities for patients with diseases afflicting the CNS.

## Data availability statement

The raw data supporting the conclusions of this article will be made available by the authors, without undue reservation.

## Ethics statement

Written informed consent was obtained from the individual(s) for the publication of any potentially identifiable images or data included in this article.

## Author contributions

MD: Writing – original draft. ARG: Writing – original draft, Writing – review & editing. MG: Resources, Writing - Reviewing and Editing, Validation. AG: Writing – original draft. ML: Writing – original draft, Writing – review & editing.

## Glossary

CSF: Cerebrospinal fluid
CNS: Central nervous system
I/O: Input/output
ICP: Intracranial pressure
NPH: Normal pressure hydrocephalus
VRS: Virchow–Robin spaces
AQP: Aquaporin
MRI: Magnetic resonance imaging
LP: Lumbar puncture
LD: Lumbar drain
RBC: Red blood cells
NfL: Neurofilament light chain
pNFH: Phosphorylated neurofilament heavy chain
NSE: Neuron-specific enolase
GBS: Guillain–Barré syndrome
TNF: Tumor necrosis factor
MSCs: Mesenchymal stem cells
